# Depression drives perceived quality of life following minor stroke

**DOI:** 10.1186/s41687-025-00861-w

**Published:** 2025-03-11

**Authors:** Martina Gjyzari, Elisabeth Breese Marsh

**Affiliations:** 1 Department of Neurology, Johns Hopkins University School of Medicine, Baltimore, MD USA; 2 Johns Hopkins Hospital, 600 North Wolfe St. Phipps 446C, Baltimore, MD 21210 USA

**Keywords:** Stroke, Recovery, Depression, Outcomes, Quality of life

## Abstract

**Background:**

Stroke outcomes are typically assessed using objective scales focused on severity and functional ability that may overlook subtle cognitive changes and fail to account for patients’ perceptions of recovery and quality of life. This study aimed to compare patient-reported outcomes (PROs) to objective recovery metrics in patients with minor stroke and identify factors associated with perceived recovery and quality of life.

**Methodology:**

Data from 134 patients with minor stroke were prospectively collected at 1-, 6-, and 12-months post-infarct. Objective assessments measured stroke severity, functional outcomes, activities of daily living, and global cognitive function. PROs included assessments of function, depression, fatigue, symptomatic improvement, and quality of life. Regression models were used to evaluate the relationship between subjective PROs and physician-obtained measures.

**Results:**

Analyses revealed an important role for mental health factors in subjective measures of recovery, though cognitive dysfunction was not significantly associated with either subjective improvement or quality of life despite being commonly endorsed. Depression and fatigue were inversely associated with both satisfaction and quality of life, along with stroke severity and overall functional impairment during both short- and long-term recovery periods. The impact of depression on quality of life increased over time, while stroke severity and functional status were associated with perceived symptomatic improvement at all time points.

**Conclusions:**

For patients with minor stroke, depression is negatively associated with perception of symptomatic recovery and quality of life, particularly at later time points. Addressing post-stroke depression may improve patient-reported outcomes, though further research is needed to determine its impact on broader measures of post-stroke morbidity.

**Supplementary Information:**

The online version contains supplementary material available at 10.1186/s41687-025-00861-w.

## Introduction

Stroke is the second leading cause of death globally. It impacts roughly 13.7 million people, resulting in 5.5 million deaths per year [1]. Patients experience a variety of symptoms depending on the location of their infarct including motor weakness, language deficits, and cognitive impairment affecting attention, concentration, and processing speed [2]. The traditional metrics used to assess stroke recovery typically evaluate both stroke severity and functional ability, and are administered on presentation to the hospital and during follow-up clinic visits. Some of these metrics include the modified Rankin Score (mRS; measures functional ability), the National Institute of Health Stroke Scale (NIHSS; measures stroke severity), and the Barthel Index (BI; measures activities of daily living) [3]. These scales are heavily weighted toward the physical manifestations of stroke and are coarse and potentially insensitive to relatively small changes especially with respect to cognition, an increasingly recognized consequence of stroke with profound effects on the ability to return to work and reintegrate into society [4]. Furthermore, these scales do not evaluate the patient’s perception of their recovery or their satisfaction and quality of life (QoL). Therefore patient-reported outcome (PRO) measures which include questionnaires evaluating perception of symptomatic improvement, satisfaction, and QoL are increasingly used in conjunction with objective scales for a more comprehensive approach [5].

The relationship between objective, physician-measured functional recovery and PROs has not been well studied, particularly over time. While it seems intuitive those with “better function” would be “happier”, factors such as depression and fatigue may complicate this relationship [6], and for many it may change over the course of their recovery as they progress through the stages of grief associated with a traumatic event [7]. By identifying factors most closely associated with their perception of symptomatic improvement and QoL, we hope to identify modifiable factors that can be leveraged to optimize outcomes at each stage of recovery (acute, subacute, chronic). In this study, we compare objectively obtained clinical performance to PROs longitudinally over the first year of recovery in a cohort of patients with minor stroke. Patients with minor stroke have the potential for an excellent long-term outcome given their small stroke size and relatively “mild” objective symptoms. However, in clinical practice we see many individuals unable to return to work and unhappy with their recovery due to cognitive and mood disorders [8]. Therefore, this population was chosen to allow the study to focus on the impact of post-stroke cognition, fatigue, and mood disorders, and fully evaluate the relationship between objective testing and PROs, avoiding the confounding effects of severe language or motor impairment.

## Methods

All patients who are admitted to the Johns Hopkins Bayview Medical Center with an acute ischemic stroke are scheduled for follow-up in the Bayview Stroke Recovery Clinic 4–8 weeks after hospital discharge. At their first follow-up visit, patients with minor stroke (defined as an admission NIHSS score < 8 to avoid significant hemiparesis or aphasia but include subcortical lacunar infarcts and small embolic lesions) are consented and prospectively enrolled in a longitudinal study evaluating recovery of minor stroke. They return 6- and 12-months post-infarct for subsequent evaluation. At each assessment, patients are administered a battery of neurocognitive tests (described below) along with PRO questionnaires evaluating depression (PHQ-9) [9], fatigue (FACIT) [10], and perception of their own functional recovery (Stroke Impact Scale) [11]. Additional information regarding: patient demographics (age, sex, self-identified race, handedness, education, occupation level [ranging from unskilled to professional]), social support (living with someone at home), baseline status (premorbid mRS and IQ [12]), medical history (history of dementia, depression, hypertension, hyperlipidemia, diabetes, smoking), stroke characteristics (NIHSS score on admission and discharge, stroke volume, impacted hemisphere, amount of white matter disease [13], etiology [14], and type of rehabilitation post‐discharge (inpatient, home, outpatient, and none) were collected as potential confounding variables. Patients lost to follow-up were censored in the analysis. Perception of symptomatic recovery and QoL are collected using Likert scales (0–7) with higher values indicating a more positive response. All data are entered into an IRB-approved, HIPAA compliant registry. The PI had access to all the data in the study and assumes responsibility for its integrity and the data analysis.

### Cognitive testing and objective assessment

Each patient also undergoes a complete neurological assessment. Functional abilities are assessed using the modified Rankin scale (mRS) and Barthel Index (BI), while global cognitive function is assessed using the MoCA (Montreal Cognitive Assessment).

### Analysis

Statistical analyses were performed using STATA version 15. Our two primary outcome variables of interest included the PROs: “patient perception of symptomatic improvement” and “satisfaction with QoL”- defined as continuous variables, measured using a Likert scale of 0–7. Univariate linear regression was used to evaluate the association between the primary outcomes and performance on objective assessment, specifically: the MoCA, mRS, BI, and NIHSS. Other PROs (SIS, PHQ-9, FACIT) were evaluated similarly, along with potential confounders (chosen a priori) including age, sex, race, education, occupation, and social support. Multivariable regression models were then used to adjust for confounders. Due to the continuous nature of our primary outcomes, which were measured using a Likert scale, linear regression was chosen as the primary approach to preserve variability, avoid arbitrary cutoffs, and allow for direct interpretation of effect sizes over time. However, logistic regression was also used to investigate the effect of stroke severity (NIHSS), functional status (mRS), fatigue (FACIT) and depression (PHQ-9) on a dichotomized QoL and perceived symptomatic recovery variable (defined as “good” above a threshold of 3). Linear regression models are presented in Table 3, and odds ratios for selected outcome variables in Table 4. Initial hypotheses predicted that stroke severity and better cognitive performance would be associated with a more favorable perception of symptomatic improvement and higher satisfaction with QoL; and that individuals with higher levels of education and socioeconomic status would report better outcomes. However, we explored the alternative possibility that expectations would be higher for those with a higher pre-morbid baseline and thus more difficult to successfully achieve. Analysis was performed independently for the for the 1-, 6-, and 12-month time points. P-values of < 0.05 were considered statistically significant.

### Data availability

The data is available from the PI upon request.

## Results

134 patients with minor stroke were admitted between November 2016 and July 2021. The average age was 64 years; 51% were women; 28% were black. On average, enrolled patients had 13.2 years of education and 15% had a prior history of depression. Full patient demographics are described in Table 1. Of the 134 patients enrolled, 76 (56.7%) were lost to follow-up by the 12-month assessment. Dropout was marginally associated with age (*p* = 0.060), with older patients more likely to be lost to follow-up. No significant associations were found between dropout and baseline depression (PHQ-9), cognitive function (MoCA), stroke severity (NIHSS), or functional disability (mRS). Individuals missing baseline QoL data were significantly more likely to be lost to follow-up.

**Table 1:** Patient demographics

Variables		Total cohort
*Demographics*		
Age, mean (SD), years		64.2 (13.8)
Education, median (IQR), years		12 (3)
Sex, n^†^ (%), male		65 (48.87)
Race, n (%), Black		37 (27.82)
Social support, n (%), yes		118 (90.08)
Handedness, n(%), right		110(85.94)
Prestroke mRS, median (IQR), points		0 (0)
Premorbid IQ, mean (SD), points		106.4 (11.18)
Occupation class code		
1 – professional, n (%)		13 (11.82)
2 – intermediate, n (%)		19 (17.27)
3 – skilled, n (%)		26 (23.64)
4 – semiskilled, n (%)		33 (30)
5 – unskilled, n (%)		17 (15.45)
Rehabilitation		
None, n (%)		34 (28.10)
Inpatient, n (%)		27 (22.31)
Home, n (%)		28 (23.14)
Outpatient, n (%)		32 (26.45)
*Medical history*,* n (%)*		
Smoking		34 (25.56)
Depression		20 (15.38)
Dementia		2 (1.53)
Diabetes Mellitus		49 (36.57)
Hypertension		104 (77.61)
Hyperlipidemia		91 (67.91)
*Stroke characteristics*		
Hemisphere, n (%), right		67 (50)
Admission NIHSS, median (IQR), points		2 (3)
Discharge NIHSS, median (IQR), points		1 (3)
White matter disease, mean CHS (SD) points		2.65 (1.21)
Stroke volume, mean (SD), cc		5.29 (11.18)
Etiology		
1 – Large artery atherosclerosis, n (%)		40 (30.08)
2 – Cardioembolism, *n* (%)		19 (14.29)
3 – Small vessel occlusion, *n* (%)		56 (42.11)
4 – Other determined etiology, *n* (%)		8 (6.02)
5 – Undetermined etiology, *n* (%)		10 (7.52)

SD: Standard Deviation

†: number

### Confounding factors

In univariate analyses, none of the a priori variables of interest were significantly associated with perception of symptomatic recovery or QoL (Table 2). Multivariable regression analyses are displayed showing the impact of confounders on objective tests and PROs (Table 3) as well as the impact of other medical factors and stroke characteristics without a significant change in results.

**Table 2:** Linear regression results for demographic variables

Primary outcome variable	Quality of life regression coefficient						Likert symptoms regression coefficient					
	1 Month		6 Month		12 Month		1 Month		6 Month		12 Month	
	*	*P*-Val	*	*P*-Val	*	*P*-Val	*	*P*-Val	*	*P*-Val	*	*P*-Val
Sex	0.396	0.292	-0.037	0.931	-0.350	0.469	0.202	0.535	-0.007	0.986	-0.279	0.509
Age	-0.005	0.715	0.003	0.981	-0.017	0.284	0.006	0.586	0.003	0.832	-0.010	0.472
Occupation	-0.092	0.561	-0.043	0.799	0.208	0.251	0.038	0.781	0.026	0.870	0.103	0.517
Education	0.556	0.458	0.089	0.256	0.053	0.524	-0.049	0.447	-0.005	0.945	0.068	0.349
Race	0.811	0.066	0.196	0.714	0.712	0.190	0.175	0.655	0.075	0.882	0.182	0.705
Ethnicity	1.866	0.169	1.079	0.286	1.097	0.487	1.530	0.185	1.166	0.225	0.976	0.480
Social support	1.074	0.089	0.105	0.867	0.529	0.387	0.556	0.303	0.400	0.499	-0.250	0.641
Rehabilitation	-0.325	0.054	0.028	0.888	-0.022	0.926	-0.214	0.142	-0.141	0.457	-0.045	0.831

*: Coefficient

**Table 3:** Multivariate regression models adjusting for confounders

Primary outcome variable	Quality of life adjusted Regression coefficient						Likert symptoms adjusted Regression coefficient					
	1 Month		6 Month		12 Month		1 Month		6 Month		12 Month	
	*	*P*-Val	*	*P*-Val	*	*P*-Val	*	*P*-Val	*	*P*-Val	*	*P*-Val
*Medical History*												
White matter disease	0.065	0.765	0.083	0.714	-0.072	0.761	0.176	0.359	0.265	0.220	-0.124	0.568
Depression	-0.556	0.281	0.013	0.981	0.506	0.352	0.167	0.715	-0.157	0.772	0.371	0.449
Handedness	-0.582	0.352	-0.560	0.468	-0.704	0.385	-0.456	0.396	-0.448	0.542	-0.626	0.390
Comorbidities	-0.194	0.236	0.082	0.643	-0.202	0.354	-0.372	**0.009**	-0.036	0.834	-0.032	0.875
Hemisphere	-0.308	0.483	-0.127	0.811	0.854	0.143	-0.431	0.265	-0.249	0.623	0.318	0.550
Hypertension	-1.059	**0.035**	0.296	0.663	-0.800	0.206	-1.039	**0.018**	0.574	0.360	-0.747	0.189
Hyperlipidemia	0.081	0.853	0.650	0.212	0.106	0.855	-0.453	0.240	0.408	0.412	0.510	0.323
Diabetes Mellitus	0.250	0.571	0.697	0.188	-0.149	0.794	-0.078	0.846	0.256	0.616	-0.388	0.447
Smoking	-1.014	**0.028**	-0.309	0.601	-0.306	0.635	-0.572	0.176	0.113	0.841	-0.601	0.297
*Stroke Characteristics*												
Prior stroke	0.142	0.808	0.344	0.594	-0.344	0.588	0.179	0.735	0.274	0.658	-0.709	0.210
Subcortical	-0.691	0.093	-0.338	0.512	-0.541	0.281	-0.949	**0.009**	-0.422	0.382	-0.300	0.509
Stroke etiology^†^	-0.039	0.829	0.129	0.556	0.259	0.235	0.001	0.994	0.130	0.535	0.206	0.296
Prestroke mRS^‡^:	-0.616	0.408	-3.623	**0.050**	0.332	0.840	-0.817	0.208	-4.117	**0.022**	-0.067	0.964
Rehabilitation	-0.165	0.367	0.172	0.454	-0.041	0.870	-0.078	0.633	-0.017	0.939	-0.032	0.886
*Functional Outcomes*												
NIHSS^§^	-0.588	**< 0.001**	-0.249	0.150	-1.058	**0.013**	-0.496	**< 0.001**	-0.348	**0.035**	-0.999	**0.031**
BI^||^	0.041	0.150	-0.033	0.729	Omitted		0.055	**0.027**	0.038	0.675	Omitted	
mRS^‡^	-0.744	**< 0.001**	-0.635	**0.014**	-1.131	**0.004**	-0.579	**0.001**	-0.672	**0.006**	-1.297	**0.002**
MoCA^#^	-0.082	0.296	-0.116	0.194	-0.029	0.778	-0.047	0.506	-0.133	0.116	-0.038	0.682
FACIT^**^	0.076	**< 0.001**	0.065	**0.002**	0.069	**0.020**	0.066	**< 0.001**	0.069	**0.001**	0.057	**0.033**
PHQ9^‡‡^	-0.145	**< 0.001**	-0.134	**0.004**	-0.181	**0.006**	-0.098	**0.002**	-0.114	**0.011**	-0.119	**0.050**
SIS^§§^	0.050	**0.001**	0.069	**< 0.001**	0.078	**< 0.001**	0.043	**< 0.001**	0.064	**< 0.001**	0.074	**< 0.001**

**Statistically significant values in bold**

*: Coefficient

†: TOAST classification

‡: Modified Rankin Score

§: NIH Stroke Scale

||: Barthel Index

#: Montreal Cognitive Assessment

**: Functional Assessment of Chronic Illness Therapy

‡‡: Patient Health Questionnaire – 9

^§§^: Stroke Impact Scale

### Quality of life

The relationship between objective scales and PROs are listed in Tables 3 and 4. The Barthel Index (BI) was omitted in the 12-month multivariate analysis due to collinearity. At 1 month post stroke, along with stroke severity (NIHSS = -0.62, *p* < 0.001) and poor functional status (mRS = -0.74, *p* < 0.001), fatigue (FACIT = 0.07, *p* < 0.001) and depression (PHQ-9 = -0.15, *p* < 0.001) were significantly associated with satisfaction with QoL, with higher scores correlating with lower QoL. While trends remained, the NIHSS lost significance at 6 months, but was again significant at 12 months. However, mRS, depression and fatigue remained significant at all time points. Depression demonstrated not only the strongest clinical significance (PHQ-9 = -1.18, *p* = 0.006), but the greatest increase in significance at 12 months. Patient perception of function demonstrated by the SIS was also significant at all time points, with better outcomes corresponding to higher QoL reports. Despite endorsing cognitive dysfunction, MoCA scores were not associated with QoL at any time point.

**Table 4:** Logistic regression models adjusting for confounders

Primary outcome variable	Quality of life odds ratio						Likert symptoms odds ratio					
	1 Month		6 Month		12 Month		1 Month		6 Month		12 Month	
	OR	*P*-Val	OR	*P*-Val	OR	*P*-Val	OR	*P*-Val	OR	*P*-Val	OR	*P*-Val
NIHSS^§^	0.817	0.196	0.807	0.299	0.201	0.077	0.710	0.076	0.884	0.644	0.201	0.077
mRS^‡^	0.757	0.242	0.459	**0.031**	Omitted		0.678	0.179	0.640	0.277	Omitted	
FACIT^**^	1.086	**< 0.001**	1.076	**0.018**	1.083	0.183	1.083	**0.002**	1.091	0.240	0.951	0.648
PHQ9^‡‡^	0.834	**< 0.001**	0.868	**0.027**	0.763	**0.030**	0.910	**0.036**	0.888	0.079	1.037	0.870

**Statistically significant values in bold**

OR: Odds Ratio

‡: Modified Rankin Score

§: NIH Stroke Scale

**: Functional Assessment of Chronic Illness Therapy

‡‡: Patient Health Questionnaire – 9

### Symptomatic recovery

Similarly, stroke severity (NIHSS = -0.50, *p* < 0.001), decreased functional ability (mRS = -0.58, *p* = 0.001 and BI = 0.06, *p* = 0.027), fatigue (FACIT = 0.07, *p* < 0.001), and depression (PHQ-9 = -0.10, *p* = 0.002) were inversely associated with perception of symptomatic improvement 1-month post stroke, along with the PRO SIS. Results remained consistent at later time points (both 6 and 12 months), but unlike with QoL, stroke severity and overall functional status rather than depression were most strongly associated with patient reports.

## Discussion

While there is a relationship between objective functional measures (NIHSS, BI, and mRS) and patient-reported outcomes, the association varies across different time points and outcomes. Initially we hypothesized that factors such as baseline function, age, education, and occupation may significantly drive this relationship, but we did not find such associations. Our data suggest that, along with objective measures such as stroke severity and functional ability, depression and fatigue are major drivers of a patient’s perception of their symptoms and satisfaction with their QoL. This appears consistent across time points, though the effect of depression on subjective QoL appears to increase during the chronic phase of recovery, becoming as important as stroke severity over time. Importantly, while depression also influences how patients report their symptomatic recovery, objectively measured stroke severity and functional status appear more strongly associated with perception of symptomatic improvement. Interestingly, despite many patients with minor stroke endorsing symptoms of cognitive impairment and scoring below average on tests of cognitive function, objective evidence of impairment on the MoCA was not related to either PRO [8].

Stroke severity and functional abilities were related to both perception of symptomatic recovery and QoL at all time points. It should not be surprising that those with lower objective function would perceive themselves to have greater symptoms and poorer QoL. This was observed using both objective testing (NIHSS and mRS) and PROs (SIS) [15]. Importantly, we did not find that this was offset by a higher premorbid baseline resulting in unrealistic expectations as we had originally hypothesized. It is also reasonable that functional status would be a significant factor for those with smaller, less severe strokes. Even those with “relatively mild deficits” can exhibit a spectrum of stroke severity. Remarkably, functional status appeared to greatly influence the way in which patients perceived their symptomatic recovery, but to a lesser degree their QoL at later time points. This may be due to an increased ability in patients to quantify their deficits more objectively as they learn to compensate for them. Conversely, mood and fatigue continue to be strong influencers, especially of QoL, in chronic stages.

Previous literature has focused heavily on motor weakness and language deficits, but functional impairment can affect many domains and result in varying levels of independence for patients. Cognition has been increasingly recognized as an important marker of function that is often impaired, particularly in those with minor stroke [16, 17]. It was therefore somewhat surprising that MoCA scores were not significantly associated with PROs at any time point. This may be because the MoCA is only a global cognitive screen rather than a more in-depth assessment of impairment, or that functional scales captured the effect of impaired cognition more fully than the cognitive screen alone. Lower MoCA scores have been directly associated with higher mRS values [18]. Many individuals exhibit their most significant cognitive impairment early on during recovery [16, 17], the same time period when functional scales were most impactful on perception.

While previous retrospective analyses suggested that the physical health effects of stroke may be stronger than the psychological effects [19] our data indicated differently. They suggest that depression and fatigue are significant drivers of both how patients perceive the recovery of their symptoms and QoL at all time points. Fatigue is strongly associated with patient perception both in the acute and subacute setting. Depression, on the other hand, most strongly impacts how patients perceive the recovery of their QoL, rather than their symptoms. This dichotomy is not surprising, as symptom perception is more closely related to functional status and seems to be driven majorly by stroke severity and overall function (BI score). However, it is notable that depression is a significant driver of perceived QoL, and this association becomes stronger over time. One possible reason for this trend is that in the subacute setting some recovery of symptoms has occurred, therefore the association between depression and perceived QoL is not overshadowed by symptom severity.

Depression has been found to be an important player in patients’ recovery after stroke in the acute, subacute, and chronic setting [20–22]. The relationship between depression and perception of recovery has been observed in different neurological populations such as the study by O’Brien et al. who found that **“**correlational analyses indicate the MSNQ-S is significantly correlated with mood and self-reports of functioning, but not with objectively measures daily functioning and to only few neuropsychological tests” [23]. Our data not only corroborate the significance of depression in recovery, but further explore this relationship by showing that depression affects perceived QoL far more than perceived symptomatic recovery.

Our findings are highly relevant to clinical care because accounting for patients’ subjective experiences invites them to become part of their care and offers an increased level of autonomy. If patients are satisfied with how they are recovering, they are more motivated to take an active role in getting better through physical and occupational therapy, taking their medications, and going to therapy. By acknowledging the mental health struggles that exist and the role they have in patients’ recoveries, providers are better prepared to offer help. This extends beyond the acute setting as there is evidence that even 1–3 years after discharge from the hospital, stroke survivors continue to suffer from depression and may benefit from a long-term intervention to counter such psychological distress [24].

This study is not without limitations that are important to consider when interpreting the results. This study was conducted at a single, urban, academic stroke center and enrolled a relatively small cohort of patients with minor stroke which might affect the generalizability, particularly with respect to those with more severe deficits. A cohort with relatively low stroke severity was chosen to avoid the potential confounding effects of aphasia and severe hemiparesis, in order to focus on the role of cognitive dysfunction in patients with relatively good function otherwise. Due to the small cohort size, multivariable regression models that adjust for key covariates were utilized rather than stratified analyses, as further stratification could reduce statistical power and limit generalizability. Thus, there exists a potential for subgroup differences that will need to be explored in future studies with larger cohorts. In addition, patient drop out over time was also significant with only 43% of patients completing their 12-month follow-up. To address whether attrition may have affected the time-dependent results of the study we compared patients with complete follow-up to those lost to follow-up. Our analysis revealed that age was the only factor even marginally associated with likelihood of follow-up (*p* = 0.060), with older patients more likely not to return. Other baseline clinical variables—including depression (PHQ-9), cognitive function (MoCA), stroke severity (NIHSS), and functional disability (mRS)—were not significantly associated with dropout. However, we did observe a slight trend toward higher dropout rates among patients with higher NIHSS scores at baseline.

Notably, patients missing QoL data at baseline were significantly more likely to drop out at later time points. This pattern extended to other subjective measures, such as PHQ-9 where individuals with incomplete baseline data were more likely to be lost to follow-up. This suggests that baseline engagement was a key determinant of retention, however it does not necessarily mean that attrition biased clinical outcome trajectories. The observed associations between QoL, perception of recovery, and clinical outcomes at later time points remained robust, as dropout was not significantly associated with stroke severity, cognitive function, or other major clinical variables and none of the baseline factors that varied between those who followed up and those who were lost were associated with perception of QoL.

Despite these limitations, this study is important because it not only illustrates the dissociation of objective clinical findings with PROs but provides a potential therapeutic target to ultimately reduce post-stroke morbidity and improve QoL long-term.

In conclusion, our findings support the anecdotal observation that a patient’s perception of their recovery does not always correlate with the objective clinical data. In addition, it identifies potentially modifiable factors such as depression as being as important as stroke severity when considering how satisfied an individual is with their outcome, especially long-term. Further research is needed to determine if more aggressive treatment of depression, particularly at later stages of recovery, will truly improve PROs as well as the ability to generalize results to those with more severe language and motor deficits.

## Electronic supplementary material

Below is the link to the electronic supplementary material.

Supplementary Material 1

## Data Availability

The datasets used and/or analyzed during the current study are available from the corresponding author on reasonable request.
