# Supplementary material for: Depression drives perceived quality of life following minor stroke
Source: J Patient Rep Outcomes. 2025 Mar 11;9:29. doi: 10.1186/s41687-025-00861-w (PMC11896911; doi:10.1186/s41687-025-00861-w)
Supplement: Supplementary file 1 — Supplementary Material 1 [file 41687_2025_861_MOESM1_ESM.pdf]

Do you agree with these statements about your recovery?

My stroke symptoms have recovered well.

Strongly Disagree ---1---2---3---4---5---6---7--- Strongly Agree

I am satisfied with my recovery/quality of life.

Strongly Disagree ---1---2---3---4---5---6---7--- Strongly Agree

The biggest factor currently impacting my quality of life is…

____

____

____

____

____

We care about your inpatient experience. How was your hospitalization?

How satisfied were you with your care?

Strongly Dissatisfied ---1---2---3---4---5---6---7--- Strongly Satisfied

Comments

____

____

____

____

____
